# Hospital Progress and Finance

**Published:** 1924-08

**Authors:** 


### HOSPITAL PROGRESS AND FINANCE.

### THE SCOTTISH HOSPITAL INQUIRY.

The Scottish Board of Health has appointed a committee to inquire into "the extent and nature of the inadequacy of the present hospital and auxiliary services in Scotland" and to make recommendations for their "development and maintenance." Lord Mackenzie is chairman, and Mr. Niven McColl is secretary of the committee, which includes Col. D. J. Mackintosh and representatives of the hospitals, insurance committees, the public health services, and Labour organizations. The subject of the inquiry appears to be wider in scope than that which is now being examined by the Voluntary Hospitals Commission in England, and it will be instructive to see from the reports of the two inquiries how far the difficulties in the two countries are the same, and how far the recommendations vary. No type of institution appears to be excluded from the Scottish inquiry, and the result must, therefore, take the form of proposals for a complete hospital system for Scotland. On both sides of the Border there are many who believe that if only full advantage were taken of the beds already in existence the scarcity of accommodation would be much less than appears. Consequently the common problem is one of creating an adequate hospital service out of the voluntary and Poor Law institutions. The Scottish Committee will be able to consider this frankly. The inquiry in England concerns the voluntary hospitals alone.

### A VITAL REBUILDING SCHEME.

The scheme for rebuilding the Kensington, Fulham and Chelsea General Hospital on its present site has been sanctioned by King Edward's Hospital Fund, and the governors have resolved to carry the scheme through. Sir Aston Webb, P.R.A., has drawn the plans, which provide for 100 beds, an out-patient department, X-ray and electrical installations, at a cost of some £50,000. We trust that this is the beginning that will at last realise the ambitions of the hospital, whose difficulties have been exceptional, long-standing and severe. The additions of 1905 left the old, unhappy trait unaltered, and concealed the improvements behind it. This part remained the symbol of all that it was desired to remove, and no effort should now be spared to bring the institution up to date. Perhaps no London hospital has had greater difficulties to overcome, and the present is a great opportunity. Those who recognise it and help to carry the scheme through will be remembered with honour, for the success of the scheme, so long overdue, will for the first time enable the institution to take its rightful place among the hospitals of London.

### SUN TREATMENT IN BERMONDSEY.

On the suggestion of its medical advisers, who have visited Switzerland to investigate upon the spot, the Bermondsey Borough Council has decided to adopt, so far as possible, the sun treatment for tuberculous patients in the borough. The report from Dr. King Brown, medical officer of health, submitted by the Public Health Committee, stated that Chailey, Alton and Carshalton, where the treatment is being carried on, would also be visited, and that a statement of the cost and certain proposals would be presented later. The Council witnessed a demonstration of slides, and Dr. Salter stated that 250 patients were sent away from Bermondsey every year to institutions, and of these 200 relapsed after their return. While the Council intended to use such sun as there was in Bermondsey, this would be supplemented by artificial sunlight. The Council thanked the doctors for their explanations, approved the Committee's action and a propaganda scheme. This is hopeful and encouraging, and one of its results must be to strengthen the absurdly half-hearted support at present given to all proposals to abate the smoke nuisance in our towns. When natural sunlight could be had for the asking, the general adoption of electric lamps in its place reminds the most thoughtless of what we deprive ourselves of unnecessarily. We recall the surprise with which a group of miners heard an English doctor say that the German Sheffield was a beauty spot.

### WESTMINSTER HOSPITAL REOPENED.

On July 15 Westminster Hospital was reopened after the reconstruction through which it has had to be closed for several months. The cost of the scheme, namely £70,000, has been met by public subscription, and this means that the offer of £16,000 by Sir H. M. Mallaby-Deeley on condition that a similar sum were raised by June 30 has borne excellent fruit in the three months since it was made. The reconstructed hospital starts work again with 230 beds, rejuvenated and slightly larger, to continue, without any new debt incurred, its honourable history.

### HOSPITAL BEDS IN DEVONSHIRE.

The Devon Voluntary Hospital Committee has made a report on the hospital accommodation of the county in connection with the inquiry undertaken by the Voluntary Hospitals Commission. The report states that twenty of the twenty-five hospitals in this area have no waiting list; that, at the beginning of June, 147 patients were awaiting treatment, mainly at Exeter, beside a number requiring radium treatment at Newton Abbot Hospital; and that no duplication of entries need be inferred. Out of a total number of 767 beds, an average of 480 only are occupied; the average percentage of occupied beds is about 54 in the smaller and 90 in the larger hospitals. Lack of accommodation appears to be confined to the Royal Devon and Exeter Hospital, and to the Torbay Hospital, where a new hospital is already proposed. Eight hospitals in the county have schemes for additional or more up-to-date accommodation. The Committee reports that there is no arrangement for using vacant Poor Law beds, nor does it consider this to be necessary. On the other hand, the Committee thinks that a system of transfer between the larger and the smaller hospitals would be desirable. The policy recommended is to encourage major operations at three centres, such as Exeter, Barnstaple, and Torquay, other operations in selected cottage hospitals, and to use the remaining cottage hospitals as far as possible for convalescents. There seems, then, little scarcity of beds in this area, but reason to believe that the existing beds could be more profitably used. To secure co-ordination should prove easier and less costly than to supply arrears of hospital accommodation, and if the Devon Voluntary Hospital Committee can achieve this, it will be able to point to a relatively clean sheet in this difficult matter. The report shows that the need can be met without very much trouble.

### SECRETARIAL CHANGES AT SHEFFIELD.

Mr. J. W. Robinson's successor, as Secretary and Superintendent of the Sheffield Royal Hospital, is Mr. W. H. Booth, for the past ten years secretary of the Jessop Hospital for Women, Sheffield. Mr. Booth began hospital work on leaving school, and his first appointment was at the Royal National Hospital, Ventnor. Thence he went to Pinewood, the Metropolitan Asylums Board Sanatorium, near Reading, next, we understand, to King Edward VII. Sanatorium at Midhurst. Mr. Booth has been at the Jessop Hospital since 1914, and will not begin his work at the Royal Hospital until his place at the Jessop has been filled. The Sheffield hospitals have a specially interesting period ahead of them, because of the plan to remove them bodily to the outskirts of the city, and no one could enter on administrative duties at a more absorbing time.

### THE MILLER HOSPITAL EXTENSION.

The festival dinner of the Miller General Hospital, Greenwich, held on June 26, was unusually important, since its main object was to raise as much as possible of the remaining £46,000 required for the extension scheme. Everything else is practically ready for a beginning to be made with the new ward block. This will contain seventy-eight beds divided into three wards, one of which will be for children. A new kitchen and storage department is to be provided, and, if the dinner and other means prove sufficiently successful, the first part of a nurses' home will be built. This home is planned to contain seventy-three bedrooms for the nurses and domestic staff. The whole cost is expected to be £70,000, of which £24,000 is in hand. The general financial position of the hospital is satisfactory, and if the new extension can be provided there seems little doubt that its maintenance can be secured.

### A PUBLIC LOAN TO A HOSPITAL.

The Manchester Public Health Committee proposes to borrow £6,000 in order to lend it to the Manchester Babies' Hospital At the time of writing the City Council has to approve, though the suggestion has received the sanction of the Finance Committee. If the scheme goes through the Ministry of Health will repay the loan by annual sums spread over fifteen years and the hospital will provide the yearly charges on the outstanding balance. Thus, provided the hospital continues for fifteen years, no charge on the rates will be involved, unless the Ministry of Health stops its payments, and this remote risk is to be covered by a mortgage on the hospital property. The Public Health Committee values the work of the hospital, and certain of its beds are reserved for cases treated under the Corporation's maternity and child welfare scheme. The Ministry's grant of £6,000 covers about half the cost of the proposed wards, which will accommodate eighty patients; and because this grant is to be spread over fifteen years, the hospital has asked the Corporation to advance the £6,000 upon the architect's certificates as the building advances. A similar sum is to be raised from voluntary sources. The plan thus proposes to convert a grant by instalments into a grant in full. It is an ingenious way of helping the hospital out of a difficulty, but it is a pity that the Ministry has made it necessary by spreading its help over so many years.

### SEGREGATION IN MENTAL HOSPITALS.

The foundation-stone of the new reception hospital for early mental disorders, connected with St. Andrew's Hospital, Northampton, has been laid with high hopes. The idea is that all patients in this stage, whether voluntary or certified, should be treated in a separate institution so as to avoid contact with advanced or degraded invalids. Further, patients progressing towards recovery are to complete their treatment in convalescent villas, with the same object. The new hospital, which has its own entrance and gardens, accommodates sixteen men and sixteen women, and can be extended when required. A Turkish bath is to be installed in a separate building. The opening is expected to take place in eighteen months' time. The difficulties in the way of treating mental diseases are sufficiently great without complicating them artificially; and there can be little doubt that the mixed ward for patients in varying stages of disorder has been a mistake, left far too long without remedy and recognition. The new plan is therefore welcome.

### IS THE RED CROSS COPYRIGHT?

The York Workpeople's Hospital Fund has been criticised by a local military officer for placing a red cross upon the envelopes issued by the fund in the course of its recent appeal. Presumably the objection rests upon the fact that that symbol was granted to the Order of St. John of Jerusalem in the seventeenth century. The fund bowed to its critic and substituted a nurse in uniform instead, although for more than ten years it had placed a red cross on its envelopes unquestioned. No doubt the sign is used very loosely, and has come to imply to the general public almost any hospital activity. Sooner or later it will probably be abused and some ruling have to be given. Meantime we suggest that the St. George's Cross should be reserved to the societies to which it rightfully belongs, and that the St. Andrew's or the Latin Cross be employed by other charities for the sick, since a red cross has a value to the eye not to be neglected by other hospital agencies. There are a dozen shapes of cross from which to choose, and provided the colour be kept, the sign is a convenience that can be used without infringing the prescriptive rights of the Order of St. John.

